# Non-ribosomal peptide synthase profiles remain structurally similar despite minimally shared features across fungus-farming termite microbiomes

**DOI:** 10.1093/ismeco/ycae094

**Published:** 2024-07-11

**Authors:** Robert Murphy, Mikael Lenz Strube, Suzanne Schmidt, Kolotchèlèma Simon Silué, N’golo Abdoulaye Koné, Søren Rosendahl, Michael Poulsen

**Affiliations:** University of Copenhagen, Department of Biology, Section for Ecology and Evolution, Copenhagen East, Denmark; Center for Microbial Secondary Metabolites, Technical University of Denmark, Kongens Lyngby, Denmark; University of Copenhagen, Department of Biology, Section for Ecology and Evolution, Copenhagen East, Denmark; Unité de Formation et de Recherche en Sciences de la Nature (UFR-SN), Université Nangui Abrogoua, Abidjan, Côte d’Ivoire; Station de Recherche en Ecologie du Parc National de la Comoé, Abidjan, Côte d’Ivoire; Unité de Formation et de Recherche en Sciences de la Nature (UFR-SN), Université Nangui Abrogoua, Abidjan, Côte d’Ivoire; Station de Recherche en Ecologie du Parc National de la Comoé, Abidjan, Côte d’Ivoire; University of Copenhagen, Department of Biology, Section for Ecology and Evolution, Copenhagen East, Denmark; University of Copenhagen, Department of Biology, Section for Ecology and Evolution, Copenhagen East, Denmark

**Keywords:** non-ribosomal peptides, Macrotermitinae, microbiomes, amplicon sequencing variant, operational biosynthetic unit, biosynthetic gene cluster, A domain

## Abstract

Fungus-farming termites (Macrotermitinae) engage in an obligate mutualism with members of the fungal genus *Termitomyces*, which they maintain as a monoculture on specialized comb structures. Both these comb structures and the guts of the termites host diverse bacterial communities that are believed to assist in sustaining monoculture farming through antagonist suppression. Among candidate bacteria-derived compounds serving this function are non-ribosomal peptides (NRPs), which are a highly bioactive class of specialized metabolites, frequently produced by symbionts within eukaryotic hosts. However, our understanding of specialized metabolites in termite-associated microbiomes is limited. Here we use amplicon sequencing to characterize both bacterial composition and NRP potential. We show that bacterial and NRP diversity are correlated and that the former varies more than the latter across termite host and gut and comb samples. Compositions of the two are governed by host species and sample type, with topological similarity indicating a diverse set of biosynthetic potential that is consistent with the long evolutionary history of the Macrotermitinae. The structure of both bacterial and NRP compositional networks varied similarly between guts and combs across the Macrotermitinae albeit with auxiliary termite genus-specific patterns. We observed minimal termite species-specific cores, with essentially no Macrotermitinae-wide core and an abundance of putatively novel biosynthetic gene clusters, suggesting that there is likely no single solution to antagonist suppression via specialized NRP metabolites. Our findings contribute to an improved understanding of the distribution of NRP potential in the farming termite symbiosis and will help guide targeted exploration of specialized metabolite production.

## Introduction

Symbiosis between animals and microbes are frequently mediated by the production of microbial specialized metabolites with a wide range of bioactivities, including in communication and defence [1, 2]. Specialized metabolites produced by defensive symbionts may aid in the targeted suppression of host antagonists [3–9]. A key class of specialized metabolites for this purpose are the non-ribosomal peptides (NRPs) that are common in bacteria [10] and often antimicrobial [11], including in symbioses [12–15]. Examples include herbivorous *Lagria* beetles that recruit NRP-producing bacteria to help protect their eggs from antagonistic bacteria [12, 16] and Southern Pine Beetles (*Dendroctonus frontalis*) hosting hybrid polyketide-NRP (PK-NRP) producing bacteria that suppress antagonists of their nutritional symbiotic fungus *Entomocorticium* sp. [17–19]. In soil communities of bacteria, previous work has investigated NRP production potential using targeted amplicon sequencing of adenylation (A) domains of the biosynthetic gene clusters (BGCs) encoding NRP synthases (NRPSs) [20–23]. Recent studies have found that A domain diversity is positively correlated with taxonomic diversity [21, 22] and abiotic factors [20, 21, 23]. Host-associated microbiomes are often diverse and could hold an abundance of NRPs of ecological importance [24, 25]; yet analyses of A domain diversity remain unexplored.

**Figure 1:**
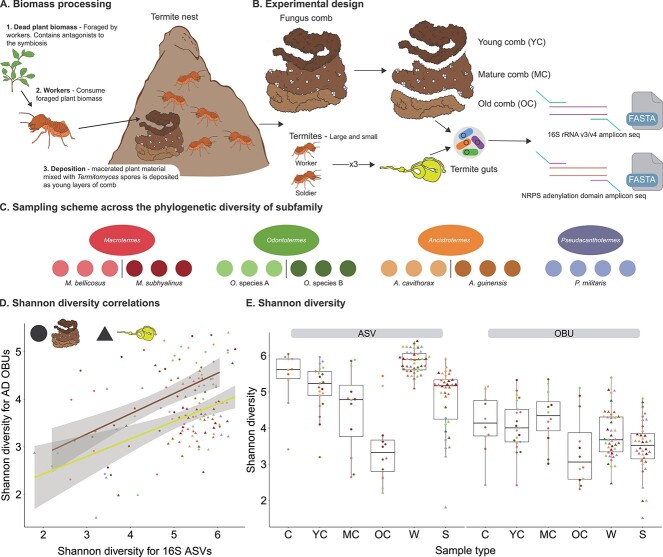
**The dynamics of fungus-farming termite biomass decomposition, experimental design and alpha diversity results. A:** A schematic representation of plant biomass decomposition in the fungus-farming termites. *Termitomyces* is cultivated in fungal combs within termite colonies, with young comb layers being formed when termites defecate a mix of *Termitomyces* asexual fungal spores and foraged plant material after an initial gut passage [97, 98]. Combs subsequently act as efficient bioreactors that, in combination with the colony microenvironment [49], provide optimal growth conditions for *Termitomyces* and full degradation of the collected plant substrates [42]. In mature layers of the fungus comb, *Termitomyces* produces nutrient, enzyme, and spore-rich nodules that serve to allow new inoculation of young comb as well as food for the termites that also ultimately eat the old comb once plant biomass is degraded [34]. **B:** Experimental workflow for sample types and sequencing approaches. **C:** We sampled 3–4 colonies for each of seven species spanning four genera in the subfamily. **D:** Correlations with 95% confidence intervals between ASV and OBU Shannon diversity for guts (triangles; lower regression line) and combs (circles; upper regression line). **E:** Alpha diversity results as exemplified by Shannon diversity indices for 16S *rRNA* ASVs (left) and a domain OBUs (right) with worker size castes and soldier size castes merged as these were not significantly different (see Fig. S1 for full version and for Chao 1 indices, and Table S2 for statistical analyses). C = mixed comb, YC = young comb, MC = mature comb, OC = old comb, W = workers, S = soldiers; data points are coloured by termite species (see C).

Termites host diverse and coevolved bacterial communities that are known to serve critical functions for their hosts [26, 27]. More so, fungus-farming termites in the termite sub-family Macrotermitinae (Termitidae: Blattodea) host both complex bacterial communities within their guts [28–30] and in their externally maintained fungus gardens (combs) [31]. In these combs, the termites cultivate a basidiomycete fungus in the genus *Termitomyces* (Agaricales: Lyophyllaceae) as an obligate lignocellulolytic partner and source of nutrition [32–34]. This symbiosis emerged some 30 million years ago and has diversified to 11 termite genera (>330 species) [35–39] and > 50 species of *Termitomyces* [40]. Complementary actions of the crop fungus and bacterial communities [28, 31, 34] enable the breakdown of plant material otherwise inaccessible to the termites [34, 41–43] (Fig. 1A). They further ensure efficient suppression of antagonists and competitors of the food fungus [44], through combined roles of termite behaviours [45–47], a carefully managed microenvironment [48, 49], and specialized metabolites [50–54]. However, only six NRPs and NPR-hybrids have been discovered in the symbiosis [54–59], leaving their diversity largely unresolved.

Farming termite gut and fungus combs have distinct properties that impact the structure and dynamics in the microbiomes they host, and hence potentially the NRP landscape. Termite guts are relatively closed, initiated by the inheritance of a large number of bacterial taxa during colony founding, followed by modulation of the microbiome until maturity [60, 61]. Ultimately, mature colonies host a diverse and consistent set of bacteria [28], dominated by Firmicutes, Bacteroidetes, Spirochaetes, Proteobacteria, and Synergistetes, with termite species-specific compositions driven by host [28], diet, and caste-specific division of labour [29, 43]. In contrast, combs are more open and, while dominated by bacterial taxa from termite gut deposits, contain microbes from the surrounding soil [31, 52, 62]. Insights into the metabolism of termite gut and comb microbiomes beyond plant biomass degradation remain sparse, but recent untargeted metabolomics have revealed a series of unknown chemical features in these environments that could include specialized metabolites [44, 63].

To improve our understanding of NRP specialized metabolites coded for by gut and comb bacteria, we sampled termites and fungus comb from seven fungus-farming termite species in Côte d'Ivoire (Fig. 1B and C). We employed targeted amplicon sequencing of the conserved A domain core genes associated with NRPS BGCs. In tandem, we amplified the V3–V4 region of the 16S *rRNA* gene to establish bacterial community compositions (Fig. 1B). We hypothesized that NRP (A domain) diversity should positively correlate with 16S *rRNA* diversity, as more taxonomic diversity should yield more specialized metabolites [21]. We further hypothesized that comb NRP profiles should mirror those of guts, as gut contents build the comb [31]. Further, comparisons across four termite genera allowed testing if termite species spanning the sub-family exhibit similar NRP profiles, despite somewhat distinct microbiomes [28, 29]. Lastly, we hypothesized that co-existence in the closed (gut) environment should lead to more interactions and greater cooperation than in the more open (comb) environment, and therefore predicted that we would find more—and predominantly positive—interactions in guts than combs.

## Materials and methods

### Sample acquisition for high-throughput amplicon sequencing

To characterize the NRPS profile across the fungus-farming subfamily, we collected material from 22 colonies from four termite genera (seven species) at the Lamto Ecological Research Station, Côte d'Ivoire in West Africa (Fig. 1B and C; Table S1). Termite species were identified morphologically and verified by barcoding of the *COII* gene based on Chelex DNA extraction as described by [64] and Polymerase Chain Reaction (PCR) as described by [65]. Nests were carefully opened and all sterile termite castes (major and minor workers and soldiers) were collected for each colony as were young, mature, and old fungus comb phases when possible. When comb phases could not be discerned, we collected a mix of comb (see Table S1 for details). Samples were stored in RNAlater® (Thermo Fisher Scientific, USA) at −20°C until DNA extractions were performed. Enough material was recovered per colony and sample type to allow three identical technical replicates per colony. Environmental control samples were collected from three sampling sites and the field station laboratory by leaving a Falcon tube with RNAlater® open to the air for 5 min.

### 16S *rRNA* and adenylation domain amplicon sequencing

The PowerSoil Pro kit (Qiagen, Germany) CD1 and CD2 buffers were used for the DNA extractions. CD1 solution (800 μl) was added to each sample type: three termite guts, ~250 mg comb, 250 μl environmental control RNAlater®, or 250 μl sterile deionized water as a control. A sample was vortexed briefly and then incubated at 65°C for 10 min. A 7 mm steel bead was introduced with sterile forceps and the sample was homogenized with a TissueLyser II (Qiagen, Germany) for 10 min at 25 Hz. A tube of G2 DNA/RNA Enhancer ceramic beads (Ampliqon A/S, Denmark) was introduced to the sample and again homogenized for 10 min at 25 Hz. The sample was centrifuged at 15 000 g for 5 min. The supernatant was transferred to a clean microcentrifuge tube, after which 200 μl CD2 buffer was added and briefly vortexed and centrifuged at 15 000 g for 5 min. Then, 700 μl supernatant was transferred to the sample holding well of a Maxwell 16 LEV Blood DNA kit cartridge, after which the Maxwell 16 Cartridge Preparation (3.C) section from the manufacturer’s protocol was followed. DNA extractions were done in triplicate for each sample. The A domain and 16S *rRNA* regions were amplified using A3F (5’-GCSTACSYSATSTACACSTCSGG-3′) and A7R (5’-SASGTCVCCSGTSCGGTA-3′) [66], and V3V4F_F (5´-CCTACGGGNGGCWGCAG-3′) and V3V4_R (5’-GACTACHVGGGTATCTAATCC-3′) primers [67], respectively, both with additional sample-specific multiplex identifier barcodes. Amplification reactions were prepared in 50 μl final volume, with 25 μl TEMPase Hot Start 2x master mix (VWR International, Søborg, Denmark), 13.6 μl RNAse free water, 3.2 μl of each primer, 1 μl of 20 mg/ml bovine serum albumin (Thermo Fisher Scientific, USA), and 4 μl template. PCR conditions were 95°C for 15 min, 35 cycles for 16S *rRNA* and 40 cycles for A domain of 95°C for 30s, 55°C for 40s and 72°C for 60s, and a final extension of 72°C for 10 min. Amplicons were cleaned with AMPure XP Reagent for PCR purification (Beckman Coulter, USA). DNA concentrations were quantified using Qubit dsDNA assays (Thermo Fisher Scientific, USA). Amplicons were pooled into 18 batches, for each primer type, of equimolar ratios and sequenced by Novogene (Cambridge, United Kingdom) on the lllumina NovaSeq 6000 250PE platform.

### Processing of 16S *rRNA* and A domain amplicon sequences

We imported raw reads into Qiime2 v2023.5 [68] as “MultiplexedPairedEndBarcodeInSequence”, where they were demultiplexed in a mixed orientation aware fashion with the Cutadapt [69] plugin *demux-paired* command. Reads were subjected to quality control, denoizing, merging, and chimera removal using the DADA2 [70] plugin as paired end for 16S *rRNA* amplicons (*dada2 denoise-paired* with trim-left-f/r: 10, trunc-len-f/r: 220) and single end for A domain amplicons (*dada2 denoise-single* with trim-left: 10, trunc-len: 220), taking only the forward reads as there was no read overlap. All 16S *rRNA* reads were trimmed by 10 bp and truncated to 220 bp according to their quality profiles. The resulting amplicon sequence variants (ASV) feature tables from differing batches were merged (*feature-table merge*) and A domain ASVs were further clustered at 95% into operational biosynthetic units (OBUs) with the VSEARCH plugin (*vsearch cluster-features-de-novo*). While clustering at 95% inevitably affects the precise number of unique ADs, it allows us to account for genetic diversity within domains and to avoid splitting features. As any potential biases from this approach will be consistent across samples, it should not affect analyses or inferences. The 16S *rRNA* amplicons were taxonomically classified by the classify-sklearn plugin using a Naïve Bayes feature classifier pre-trained on the Silvia 138 99% OTU full length sequences reference dataset [71–73]. The resulting ASV tables and taxonomy assignments were exported to the phyloseq package v1.44.0 [74] in the R v4.2.2 [75] environment in RStudio, where 16S *rRNA* ASVs assigned to chloroplast and mitochondria were removed. 16S *rRNA* ASVs (hereafter ASVs) and A domain OBUs (hereafter OBUs) found in only one sample or with <100 reads in all samples were removed. Contaminants were identified and removed using the prevalence method of the R decontam package v1.18.0 [76]. Technical replicates were merged by taking their means, and feature tables were subsequently standardized by square rooting with base R *sqrt* and for compositional analyses normalized with Wisconsin double standardization with the vegan package [77] v2.6.4 *wisconsin* function.

### Diversity indices

All analyses were performed using R v4.2.2 [75]. We calculated Chao1 richness and Shannon diversities on un-normalized feature tables using the *estimateR* and *diversity* functions in the vegan package [77], respectively. The effect of sample type and host species on diversity indices was calculated with base R *aov model* “Diversity = sample type * termite host species”, followed by pairwise comparisons using *TukeyHSD* and effect size estimation using the effect size [78] v0.8.3 *cohens_f* function. Pearson correlations between OBU and ASV Shannon diversities were obtained using *cor.test (method = “pearson”)* from base R. Slopes were determined by linear modelling of the relationship using *lm(OBU ~ ASV)* from base R.

To compare community composition between sample types and host species, we used normalized data sets and calculated Bray Curtis dissimilarities with *vegdist* from the vegan [77] package with *method = bray*. Non-metric multidimensional scaling (NMDS) ordination plots were generated with vegan’s *metaMDS* using *k = 4*, *maxit = 999* and *trymax = 500*, which were subsequently visualized with ggplot2 [79] v3.4.2. We then used Procrustes rotational analysis to determine correspondence between the topology of ASV and OBU ordinations using the *procrustes* and *protest* functions of the vegan package, considering an M2 value around 0.3 to demonstrate similar ordination shape. An Analyses of Variance (ANOVA) using base R *aov* was employed to determine if sample type or termite host genus affected the residual errors. The effect of sample type and termite host species on ASV and OBU composition was determined with Permutational multivariate analyses of variance (PERMANOVAS) using *adonis2* from vegan [77] (feature table ~ sample type * species). This relationship was interrogated further with pairwise PERMANOVAS to identify specific differences between sample types using *pairwise.adonis2* from the pairwiseAdonis v4 package (https://github.com/pmartinezarbizu/pairwiseAdonis) with the same model as for the non-pairwise comparisons. To clarify if grouping ASVs to genus would alter the relationship between composition and termite host genus / sample type, we used phyloseq v1.44.0 [74] and then rebuild the previously described models with the new feature table.

### Co-occurrence networks of respectively 16S *rRNA* ASVs and OBUs

To identify if relatively closed (guts) vs. more open (combs) sites would impact interactions and co-occurrence patterns of ASVs and OBUs, we generated co-occurrence networks using SpieceEasi [80] and pulsar [81] packages. We did this individually for each termite species and separately for gut and comb samples. Since these methods have internal standardization, we used the un-normalized count data and included only OBUs and ASVs present in at least 50% of samples for a given termite species and sample type. The Meinshausen and Bühlmann neighborhood selection method was applied with 500 repetitions. Model sparseness was inferred with the Stability Approach to Regularization Selection criterion [82]. Stability of near 0.05 [80] was achieved by adjustment of the lambda parameters. The properties of the resulting networks were investigated, including the number of associations between nodes (degree), if associations were positive or negative (weight) and how divided networks were into distinct modules (modularity). The effect of sample type and host species on degree distributions for both ASV and OBU networks was determined with an ANOVA using base R *aov* function (degree distributions ~ sample type * host species). Significant differences in modularity between sample types were identified with Student’s t tests using the base R *t.test* function.

### Core constituent and OBU novelty

Because it is conceivable that OBUs derived from BGCs of ecological relevance would be ubiquitously present in a termite species, we recovered OBUs present in 90% of all gut and comb samples for each species. We excluded soldier guts as they do not deposit faeces in the comb. We plotted OBUs identified in a given species as the logged mean normalized abundance of OBUs in that termite species and highlighted the 90% core. To determine if core OBUs belonged to BGCs with potentially similar functions, we assessed their pairwise sequence similarity by multiple sequence alignment with Clustal Omega v1.2.4 [83]. We then used PERMANOVAs in vegan [77] *adonis2* to determine associations between core OBUs and sequence similarity for all OBUs.

As the identified OBUs are likely to represent yet undescribed OBU sequences, we assessed the novelty of amplified NRP BGCs by aligning OBUs to the MIBiG v3.1 (Minimum Information about a BGC) database [84] with blastx [85] 2.14.1+ and recovered the best hit per OBU based on e-values and then removed hits with bit-scores <50 and sequence similarities <50%. To determine if core OBUs were more novel than a random sampling of a similar number of OBUs from the MIBiG database, we resampled, without replacement, 308 OBUs (the total number of species-specific core OBUs in guts and combs) 1000 times and compared the mean percent of OBUs with hits in the database with a bit-score > 50 and sequence similarity >50% to what we observed in our samples.

## Results

### Taxonomic complexity varies whilst biosynthetic potential remains consistent

Prior to pooling of triplicates and removal of low abundance and contaminant ASVs, we acquired 74 445 712 reads (159 592 per sample ± 3408). After merging and removal, we retained 19 967 957 reads assigned to 15 960 ASVs. For OBUs, we acquired a total of 6 763 249 reads (15 105 per sample ± 691) and retained 1 723 428 reads (2407 OBUs) after merging and removal. We confirmed our first hypothesis that there was a positive association between ASV and OBU Shannon diversity (Fig. 1D). Correlations were moderate for both combs (Pearson correlation: t_53_ = 4.976, r = 0.564, *P* < .001) and guts (t_76_ = 4.039, r = 0.420, *P* < .001), and for a given ASV level, OBU diversity tended to be higher in combs than guts (Fig. 1D). The substantial dispersion in ASV Shannon diversities (Fig. 1D) was, at least in part, driven by significant effects of host species (ANOVA: F_6,84_ = 2.897, *P* = .0126, Cohen’s f = 0.44) and sample type (F_8,84_ = 22.63, *P* < .001, Cohen’s f = 1.43). Conversely, host species did not affect OBU Shannon diversity (F_6,84_ = 0.938, *P* = .4719) but sample type did (F_8,84_ = 2.901, *P* = .0064, Cohen’s f = 0.51) (Fig. 1E). However, post hoc TukeyHSD testing of sample type showed no pairwise differences in OBU Shannon diversity (Table S2), suggesting, in combination with saturation of rarefaction curves (See online supplementary material Fig. S2), that while the diversity of bacterial taxa may vary, the biosynthetic potential remained fairly consistent across farming termite microbiomes. Chao1 richness estimates provided similar patterns, except that OBU richness was unaffected by sample type (See online supplementary material Fig. S1). Post hoc Tukey HSD multiple comparisons of the ANOVA model revealed that worker guts had consistently higher ASV Shannon diversity than all other sample types, except mixed comb (Fig. 1E) (Table S2). As predicted, patterns of OBU diversity in comb mirrored those of guts, consistent with combs being built from gut deposits.

### Termite host species and sample type are strong drivers of ASV and OBU compositions

**Figure 2:**
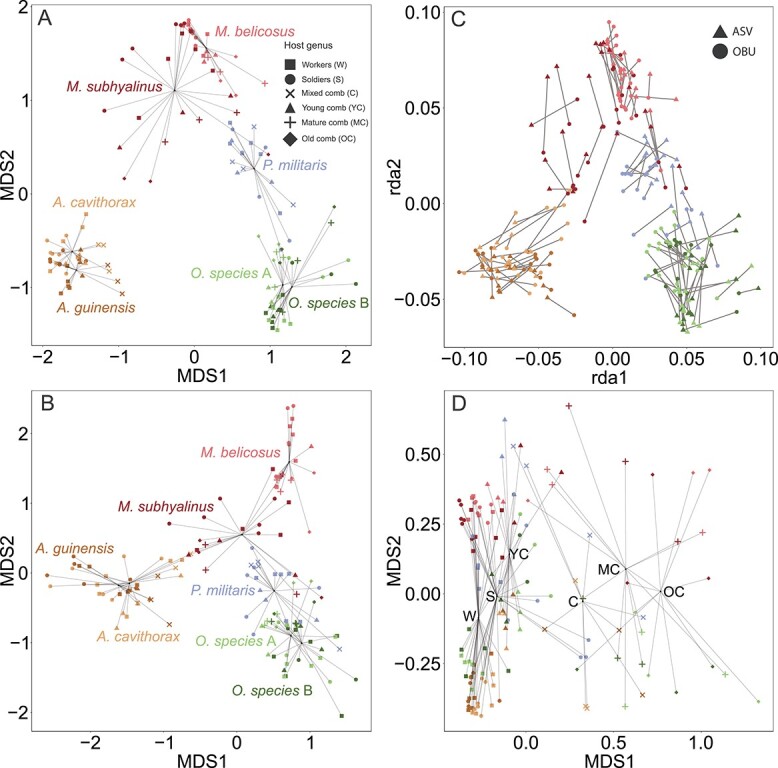
**Strong effects of termite host species generates similar patterns in ASV and OBU compositions**. **A** and **B:** NMDS ordination plots of compositional dissimilarity for ASVs (**A**) and OBUs (**B**) coloured by termite host species and labelled by sample type, showing comparable grouping by termite species and to a lesser extent sample type. **C:** Procrustes rotational analysis showing similar topology between ASV and OBU ordination. **D:** NMDS of bacterial species dissimilarity grouped by taxonomically-assigned genus, indicating an increased effect of sample type (along MDS1) compared to ASV-level comparisons (**A**) yet with ample variation within sample types across the second dimension.

We next used multivariate PERMANOVA analyses to infer how sample type and host species affected community composition, which showed that 59.5% and 68.6% of the variation in OBU and ASV composition, respectively, could be explained by termite host species, sample type, or their interaction. For species with two soldier castes, pairwise PERMANOVAs showed no differences between the two (F_1,18_ = 0.164, *P* = .937), so to avoid comparisons of species with one soldier caste (*Odontotermes*) to species with two (remaining genera), we merged soldier castes into one sample type. Host species was the main factor driving ASV (F_6,94_ = 20.75, R^2^ = 0.416, *P* < .001; Fig. 2A) and OBU (F_6,94_ = 13.51, R^2^ = 0.3491, *P* < .001; Fig. 2B) compositions, followed by sample type (ASV: F_6,94_ = 3.967, R^2^ = 0.0795, *P* < .001; OBU: F_6,94_ = 2.693, R^2^ = 0.0696, *P* < .001), which did, however, depend on host species (OBU: F_26,94_ = 1.759, R^2^ = 0.176, *P* < .001; ASV: F_26,94_ = 2.200, R^2^ = 0.191, *P* < .001). Procrustes rotational analysis confirmed that OBU and ASV ordinations were similar in topology (M^2^ = 0.308, *P* = .001; Fig. 2C), reinforcing that sample type and host species govern compositions.

Pairwise PERMANOVAs corroborated that sample type affects composition, with distinct separations between soldiers and workers indicating patterns that align with diet and division of labour, while there was no difference between different worker castes (Fig. 2A and B, Tables S3 and S4). Guts were compositionally different from all comb phases (Fig. 2A and B, Tables S3 and S4), including mixed comb where phases were indiscernible in *Pseudacanthotermes* and *Ancistrotermes*, with the exception of OBU composition of large workers to young comb (Fig. 2B, Table S3). However, compositional differences to termite guts were in general smaller for young than mature, old, or mixed comb, as evident from lower partial R^2^ values (Tables S3 and S4). Consistent with this, mature and old comb were not significantly different (ASV: F_1,16_ = 0.854, *P* = .896; OBU: F_1,16_ = 1.567, *P* = .174), but both differed from young comb (ASV: *P* < .001, R^2^ = 0.0636 and 0.0522; OBU: *P* < .0001, R^2^ = 0.0542 and 0.0396, for old comb and mature comb, respectively; Table S3).

When grouping ASV amplicon data by their putatively assigned genera, the variation explained increased from 68.6% to 75.0% and the effect of sample type on community composition increased substantially (F_6,89_ = 19.74, R^2^ = 0.316, *P* < .001, compared with F_6,94_ = 3.967, R^2^ = 0.0795, *P* < .001) while the effect of host species decreased (F_6,89_ = 16.90, R^2^ = 0.269, *P* < .001, compared to F_6,94_ = 20.75, R^2^ = 0.416, *P* < .001). The interaction between the two remained similar (F_26,89_ = 2.411, R^2^ = 0.166, *P* < .001 vs. F_26,94_ = 2.200, R^2^ = 0.191, *P* < .001). The NMDS ordination plot revealed an almost linear separation of sample type centroids along axis 1 (Fig. 2D), suggesting that phylogenetically similar bacteria are retained across the termite subfamily. Patterns of pairwise differences were similar to those of the ungrouped data, except that R^2^ values were larger in all cases (Table S5).

### Comb OBUs have more interactions than gut OBUs and they are more complementary

**Figure 3:**
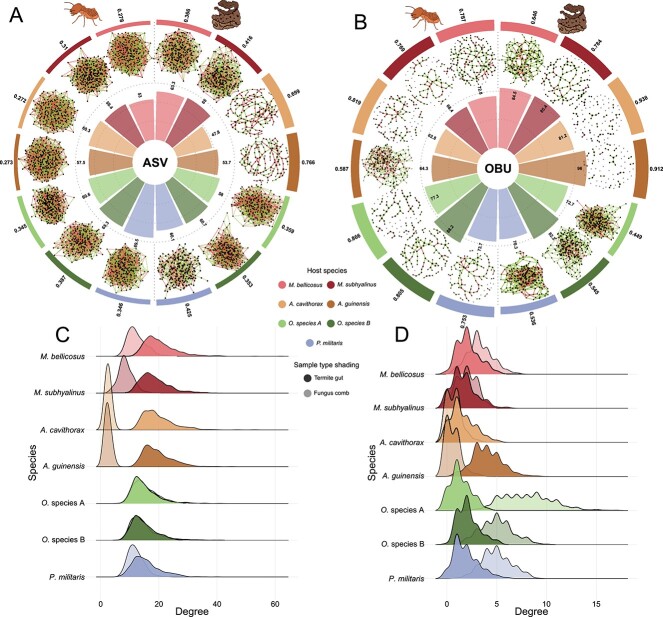
**Openness of environment is not the sole director of network structure**. **A** and **B:** ASV (**A**) and OBU (**B**) co-occurrence networks with termite gut samples on the left and fungus comb samples on the right. The networks are reduced versions, limiting nodes to only those with degree greater than or equal to the degree of the 100^th^ node in descending order. The inner bar plots show percentage edge weights that are positive, indicating that OBU networks have more positive interactions than ASV networks. OBU networks are more modular than ASV networks in guts but not fungus combs (outer bar plots). **C** and **D:** Degree distributions for ASV (**C**) and OBU (**D**) networks, indicating that termite guts have more interactions than comb samples for ASV networks while the opposite is true for OBU networks.

Co-occurrence networks for ASV and OBUs were constructed separately for gut and comb samples of a given species to test our third hypothesis that the closed or open properties of the environment affected interactions and complementarity in taxonomic and biosynthetic potential (Figs. 3A and B). Degree distributions for ASV networks were higher for gut than comb samples, indicating greater interdependence in guts (ANOVA: F_1,8916_ = 3239, p < .001) (Figs. 3A and C). The reverse was true for OBU networks (F_1,2044_ = 1167, p < .001) (Figs. 3B and D), indicating greater interdependence of NRP biosynthetic potential in combs. However, this effect was dependent on host species (ASV: F_6,8916_ = 534.2, *P* < .001; OBU: F_6,2044_ = 287.7, *P* < .001). For example, *Odontotermes* species A and B ASV networks were similar in degree distributions between sample types, while *Ancistrotermes* species were markedly higher in degree levels in guts than combs (Fig. 3C and D). Edge weight ratios indicated that associations were more positive than negative in both guts and combs for OBU but more equal for ASV networks (Fig. 3A and B). There was more variability in percentage positive associations in OBU than ASV networks for both sample types, likely due to the lower degree of OBU networks. Modularity of ASV networks was significantly higher in combs than guts (comb mean = 0.484 ± 0.173, gut mean = 0.316 ± 0.045, t-test: t_6.802_ = 2.476, *P* = .043) while the opposite was the case for OBUs (comb mean: 0.687 ± 0.193, gut mean: 0.769 ± 0.089); although this was not significant (t_8,41_ = −1.025, *P* = .334). Modularity of ASV networks were significantly lower than OBU networks in guts (t_8,89_ = −12.08, *P* < .001) but not combs (t_11,861_ = −2.065, *P* = .060) (Fig. 3A).

### Highly abundant minimal core reveal potentially important OBUs

**Figure 4:**
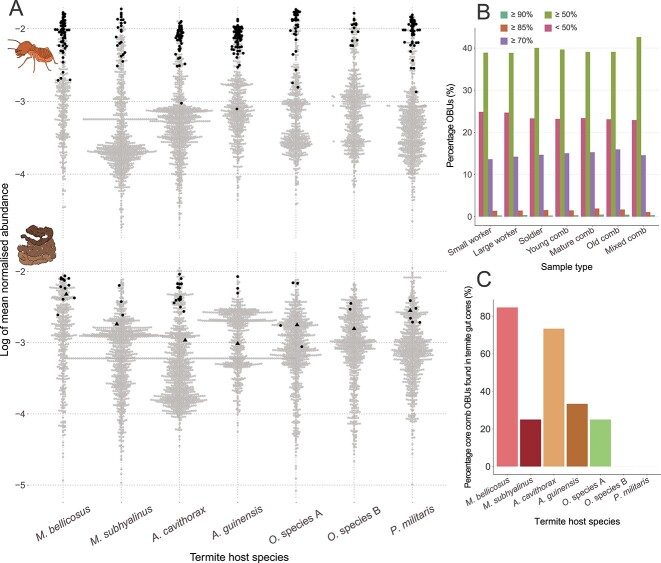
**Core OBUs are frequently abundant and disproportionate in relative abundance**. **A:** Relative abundances of OBUs by termite species, plotted as the log of the mean normalized abundance and with core OBUs (by species) highlighted in black, for guts (top) and combs (bottom). The single species wide core OBU within comb samples is indicated with an enlarged triangle. **B:** The percentage of all identified OBUs with hits to the MIBiG database grouped by sequence identity, indicating remarkably consistent patterns across sample types with few OBUs with high similarities and a large number of potentially novel OBUs. **C:** The percentage of fungus comb core OBUs that are also core in termite worker guts, suggesting variable transfer incidences of OBUs from guts to fungus combs between termite species and genera.

To identify OBUs of potential ecological relevance, we identified OBUs present in 90% of either termite worker guts or combs (the 90% OBU core). Surprisingly, only a single OBU was identified to be in the comb core, and none were identified across all worker guts. The core comb OBU across samples accounted for 0.22% of the 2403 OBUs. Given this limited overall core, we focused our search to be within host species. This revealed more core OBUs per species in the gut (14–57) than comb (2–15). Notably, all core OBUs were relatively abundant, being 3–7 times more abundant than if all were equally abundant (Fig. 4A; Table S6). Similarly, comb core OBUs were in general also relatively more abundant than other OBUs (Fig. 4A; Table S6). There was some cross-over between cores in worker guts and combs, particularly for *M. bellicosus* (84.6% overlap) and *A. cavithorax* (73.3% overlap), while this was <50% for other species (Fig. 4C). To test if core OBUs were functionally similar, we explored the relationship of pairwise sequence similarity (through K-tuple distance) between OBUs and their assignment as core, revealing no significant association (PERMANOVAs: *P* > .05).

### Novel specialized metabolite potential in the fungus-farming termite symbiosis

Our exploration for novelty through alignment of OBUs to the MIBiG database revealed extensive potential for novel NRPSs in the symbiosis: >15% of the OBUs showed <50% sequence similarity to genes in the database and < 2% had similarities of 85% or higher (Fig. 4B). Of the 308 species-specific core OBUs, 65 (21.1%) had hits in the MIBiG database with percent identities >50%. This was substantially lower than if we had randomly selected 308 of the total pool of identified OBUs (57.9%). Twelve of the 15 OBUs with sequence identities >60% aligned to BGCs coding for known antimicrobial compounds (Table S7). The best hit to the database was the comb core OBU with 79.7% sequence similarity to the BGC that encodes the synthesis of tyrocidine, which is a cyclodecapeptide known to be produced by *Brevibacillus parabrevis* [86]. We also recovered good-quality hits to NRP/NRP-PKS hybrids responsible for the synthesis of actinomycin, bacillaene, and microtermolide, which have previously been identified in the symbiosis [54, 57, 59] (Table S7).

## Discussion

Our exploration of bacterial communities and their associated NRPS biosynthetic potential from seven fungus-farming termite species revealed that bacterial richness and diversity vary by termite caste and fungus comb phase while the biosynthetic potential encoded for in these environments is more consistent. We confirmed our first hypothesis that ASV and OBU Shannon diversity are correlated, albeit with relatively weak correlations and low slopes that may indicate saturation of NRP biosynthetic potential. We further show that the underexplored fungus comb has vast potential for specialized metabolites, matching that of the termite gut microbiomes, where most previous focus has been given [50, 52, 56, 87–89]. Compositional analyses of biosynthetic potential showed distinct patterns by host species in a similar fashion to taxonomic composition, both of which were also strongly affected by termite caste and fungus comb phase. Co-occurrence networks allowed us to verify that environmental openness affects the frequency of interactions among ASVs. Specifically, higher degrees in gut ASV networks in most termite species indicate more interactions in guts than combs, while the reverse appears to be the case for OBU networks where fungus combs hold higher degrees. Associations in OBU networks were overwhelmingly positive, while ASV networks were more balanced. Modularity of networks was observed to be higher in combs than guts for ASV networks while this was not the case for OBU networks. Despite hosting taxonomically similar communities, only very few OBUs were ubiquitously shared within and especially between termite species, but they accounted for a disproportionately high relative abundance. The substantial potential for novel metabolites thus appears to be primarily NRPs that comprise core OBUs.

Our findings confirm and expand insights into species-specific and distinct gut and fungus comb microbiomes [28, 31], how termite castes structure gut communities [29, 30], and elaborate that the functional NRP biosynthetic potential follows similar patterns. The compositional similarity between young comb and termite worker guts reaffirms that comb communities are strongly driven by frequent gut deposits [31], while mature and old comb become significantly dissimilar. The distinct NRP and bacterial compositions across termite hosts imply a seemingly diverse set of NRPs across the Macrotermitinae, despite ecologically similarities, yet consistent with modifications over extended evolutionary time. The stronger effect of sample type than host species when grouping ASVs by their assigned bacterial genera support that phylogenetically similar bacterial taxa occupy termite species, suggesting long-term functional retention across the Macrotermitinae. This is consistent with prominent vertical transmission of gut microbiomes in *Macrotermes* spp. that is conserved at the genus but not ASV level [60, 61]. This also implies that very strict clustering thresholds, such as those of ASVs and OBUs, generate diversity estimates that may not be ecologically relevant. It also implies that future metagenome-predicted BGCs grouped by gene cluster families should reveal more conservative patterns in biosynthetic potential across Macrotermitinae species.

The extent of openness of the environment affected levels of bacterial interactions as predicted, causing higher degree in ASV network structures of guts (relatively closed) than combs (relatively open). The fairly balanced nature of these associations, as evident from relatively equal positive and negative edge weights, is consistent with findings from other complex microbiomes [90] and indicates both substantial co-occurrence and co-exclusion. The low modularity and high overall degree of ASV networks for guts implies high interdependence, while high modularity and lower degree in combs could imply filtering that leaves groups of ecologically similar taxa [91]. The inverse nature of closeness and degree relationship for OBU networks countered our prediction and suggests that the comb is a site with more interdependency and complementarity between NRPs than termite guts. We cannot rule out that network features could be impacted by identification of A domains on the same BGCs [92]. However, high modularity, high rates of positive interactions, and overall low degree support the presence of groups of complementary NRPs within these networks. If these are antimicrobial and act synergistically, it could mirror findings from other symbiosis [51], such as the European Beewolves, where *Streptomyces* produces a series of antimicrobial compounds to protect host larvae from fungal infections [4]. If similar complementary dynamics apply in termite fungus combs, they could act as an important site of antagonist suppression.

The majority of OBUs either had no or low-quality hits from the MIBiG database suggesting putative novelties, supporting that the symbiosis carries vast specialized metabolite discovery potential. Of the OBUs considered core within a species, which may imply ecological importance within the symbiosis, many had high-quality hits and the best of those hits frequently mapped to NRP specialized metabolites with known antimicrobial activities (e.g. [86, 93]; for a full list of references, see Table S7). Given their consistent presence within termite species, one might expect them to be present in chemical analyses. However, previous untargeted metabolomics approaches identified none of the compounds predicted based on MIBiG hits [44, 63]. The reason for this could be that low* in situ* concentrations preclude detection in complex untargeted metabolomes. Alternatively, if compounds serve specific antimicrobial functions, they may only be produced in discrete contexts and/or in specific termite castes or comb phases. This highlights the need to employ more targeted chemical analyses and detailed exploration of contexts within which BGCs are predicted to be expressed. Future work that can integrate these putative roles in the contexts of behavioural and chemical defensive means in termite hosts and the *Termitomyces* cultivar (e.g. [53]) would be particularly valuable to gain robust insights into complementary defences.

Tyrocidine putatively encoded by the comb core OBU has a broad range of antimicrobial activities [86], including antifungal activity against the fungal genus *Aspergillus* that may antagonize fungus-farming termites [94]*.* Of the specialized metabolites previously identified in the symbiosis, to which we obtained high quality hits, actinomycin and bacillaene appear selectively antimicrobial to the fungal sub-genus *Pseudoxylaria* (Xylariales: Xylariaceae: *Xylaria*) [54, 57]. *Pseudoxylaria* spp. are largely specific to termite fungus combs [95], where they exhibit a sit-and-wait strategy and only emerge once combs are compromised [96]. If actinomycin and bacillaene are critical components in the suppression of *Pseudoxylaria*, they are likely to be important across termite species. However, the small species-specific cores and the essential absence of a Macrotermitinae-wide core set of OBUs indicate that there is not a single set of defensive metabolites that act alone across the entire termite sub-family. Furthermore, it is likely that only a fraction of the chemical diversity we uncover serves defensive functions for termite hosts, but that compounds instead play roles in bacterial interactions within these complex communities. Future experimental work to improve our understanding of the roles of chemical constituents in defensive vs. beneficial or antagonistic bacterial community dynamics are needed.

While our exploration of AD domain diversity in the farming symbiosis implies ample potential for future defensive specialized metabolite discoveries, future structure elucidation and functional testing will be needed to establish ecological roles in the symbiosis. These roles are likely to not necessarily be defensive, but the persistence and diversity imply relevant roles. Further, while our work focused on exploring the diversity of putative NRPS domains, the established presence of a diversity of other compounds in the symbiosis [53], including compounds with antimicrobial properties such as polyketides, implies that our approach serves as a valuable proof of concept for similar exploratory approaches for discoveries of novel chemical diversity across compound classes in the farming termite symbioses and beyond.

## Supplementary Material

Table_S1_ycae094

Table_S2_ycae094

Table_S3_ycae094

Table_S4_ycae094

Table_S5_ycae094

Table_S6_ycae094

Table_S7_ycae094

Supplemental_Information_ycae094

## Data Availability

Amplicon sequences are available in the SRA archive in GenBank (BioProject PRJNA1082695) and barcodes for termite species verification are available from available from GenBank accessions PP986973-PP986994. Scripts generated for analysis available from https://github.com/Rob-murphys/NRPS_composition.

## References

[1] Hartmann T . The lost origin of chemical ecology in the late 19th century. Proc Natl Acad Sci USA 2008;105:4541–6. 10.1073/pnas.0709231105 18218780 PMC2290813

[2] O’Brien J , Wright GD. An ecological perspective of microbial secondary metabolism. Curr Opin Biotech 2011;22:552–8. 10.1016/j.copbio.2011.03.010 21498065

[3] Kaltenpoth M , Göttler W, Herzner G et al. Symbiotic bacteria protect wasp larvae from fungal infestation. Curr Biol 2005;15:475–9. 10.1016/j.cub.2004.12.084 15753044

[4] Kroiss J , Kaltenpoth M, Schneider B et al. Symbiotic Streptomycetes provide antibiotic combination prophylaxis for wasp offspring. Nat Methods 2010;6:261–3. 10.1038/nchembio.331 20190763

[5] Currie CR , Scott JA, Summerbell RC et al. Fungus-growing ants use antibiotic-producing bacteria to control garden parasites. Nature 1999;398:701–4. 10.1038/19519

[6] Currie CR , Bot ANM, Boomsma JJ. Experimental evidence of a tripartite mutualism: bacteria help protect leaf-cutting ant fungal gardens from specialized parasites. Oikos 2003;101:91–102. 10.1034/j.1600-0706.2003.12036.x

[7] Martín-Platero AM , Valdivia E, Ruíz-Rodríguez M et al. Characterization of antimicrobial substances produced by *Enterococcus faecalis* mrr 10-3, isolated from the uropygial gland of the hoopoe (*Upupa epops*). Appl Environ Micr 2006;72:4245–9. 10.1128/AEM.02940-05 PMC1489579 16751538

[8] Martín-Vivaldi M , Peña A, Peralta-Sánchez JM et al. Antimicrobial chemicals in hoopoe preen secretions are produced by symbiotic bacteria. Proc R Soc B 2009;277:123–30. 10.1098/rspb.2009.1377 PMC2842625 19812087

[9] Gil-Turnes MS , Fenical W. Embryos of *Homarus americanus* are protected by epibiotic bacteria. Biol Bull 1992;182:105–8. 10.2307/1542184 29304709

[10] Wang H , Fewer DP, Holm L et al. Atlas of nonribosomal peptide and polyketide biosynthetic pathways reveals common occurrence of nonmodular enzymes. Proc Natl Acad Sci USA 2014;111:9259–64. 10.1073/pnas.1401734111 24927540 PMC4078802

[11] Süssmuth RD , Mainz A. Nonribosomal peptide synthesis—principles and prospects. Angew Chem Int Ed 2017;56:3770–821. 10.1002/anie.201609079 28323366

[12] Flórez LV , Scherlach K, Gaube P et al. Antibiotic-producing symbionts dynamically transition between plant pathogenicity and insect-defensive mutualism. Nat Commun 2017;8:15172. 10.1038/ncomms15172 28452358 PMC5414355

[13] Tanaka A , Tapper BA, Popay A et al. A symbiosis expressed non-ribosomal peptide synthetase from a mutualistic fungal endophyte of perennial ryegrass confers protection to the symbiotum from insect herbivory. Mol Microbiol 2005;57:1036–50. 10.1111/j.1365-2958.2005.04747.x 16091042

[14] Partida-Martinez LP , Hertweck C. A gene cluster encoding rhizoxin biosynthesis in *Burkholderia rhizoxina*, the bacterial endosymbiont of the fungus *Rhizopus microsporus*. Chembiochem 2007;8:41–5. 10.1002/cbic.200600393 17154220

[15] Zan J , Li Z, Tianero MD et al. A microbial factory for defensive kahalalides in a tripartite marine symbiosis. Science 2019;364:eaaw6732. 10.1126/science.aaw6732 31196985 PMC12168154

[16] Dose B , Niehs SP, Scherlach K et al. Unexpected bacterial origin of the antibiotic icosalide: two-tailed depsipeptide assembly in multifarious *Burkholderia* symbionts. ACS Chem Biol 2018;13:2414–20. 10.1021/acschembio.8b00600 30160099

[17] Six DL . Ecological and evolutionary determinants of bark beetle —fungus symbioses. Insects 2012;3:339–66. 10.3390/insects3010339 26467964 PMC4553632

[18] Blodgett JAV , Oh D-C, Cao S et al. Common biosynthetic origins for polycyclic tetramate macrolactams from phylogenetically diverse bacteria. Proc Natl Acad Sci USA 2010;107:11692–7. 10.1073/pnas.1001513107 20547882 PMC2900643

[19] Cao S , Blodgett JAV, Clardy J. Targeted discovery of polycyclic tetramate macrolactams from an environmental *Streptomyces* strain. Org Lett 2010;12:4652–4. 10.1021/ol1020064 20843016 PMC2952660

[20] Geers AU , Strube ML, Bentzon-Tilia M. Small spatial scale drivers of secondary metabolite biosynthetic diversity in environmental microbiomes. mSystems 2023;8:e00724–2. 10.1128/msystems.00724-22 PMC10134846 36790187

[21] Mantri SS , Negri T, Sales-Ortells H et al. Metagenomic sequencing of multiple soil horizons and sites in close vicinity revealed novel secondary metabolite diversity. mSystems 2021;6:e0101821. 10.1128/msystems.01018-21 34636675 PMC8510542

[22] Borsetto C , Amos GCA, da Rocha UN et al. Microbial community drivers of pk/nrp gene diversity in selected global soils. Microbiome 2019;7:78. 10.1186/s40168-019-0692-8 31118083 PMC6532259

[23] Lemetre C , Maniko J, Charlop-Powers Z et al. Bacterial natural product biosynthetic domain composition in soil correlates with changes in latitude on a continent-wide scale. Proc Natl Acad Sci USA 2017;114:11615–20. 10.1073/pnas.1710262114 29078342 PMC5676913

[24] Woodhouse JN , Fan L, Brown MV et al. Deep sequencing of non-ribosomal peptide synthetases and polyketide synthases from the microbiomes of australian marine sponges. ISME J 2013;7:1842–51. 10.1038/ismej.2013.65 23598791 PMC3749504

[25] Stevenson LJ , Owen JG, Ackerley DF. Metagenome driven discovery of nonribosomal peptides. ACS Chem Biol 2019;14:2115–26. 10.1021/acschembio.9b00618 31508935

[26] Brune A , Ohkuma M. Role of the termite gut microbiota in symbiotic digestion. In: Bignell D.E., Roisin Y., Lo N. et al. (eds.), Biology of Termites: A Modern Synthe*s*is. Springer, Dordrecht, 439–75.

[27] Brune A . Symbiotic digestion of lignocellulose in termite guts. Nat Rev Micro 2014;12:168–80 10.1038/nrmicro3182 24487819

[28] Otani S , Mikaelyan A, Nobre T et al. Identifying the core microbial community in the gut of fungus-growing termites. Mol Ecol 2014;23:4631–44 10.1111/mec.12874 25066007

[29] Otani S , Zhukova M, NgA K et al. Gut microbial compositions mirror caste-specific diets in a major lineage of social insects. Environ Micr Rep 2019;11:196–205. 10.1111/1758-2229.12728 PMC6850719 30556304

[30] Li H , Dietrich C, Zhu N et al. Age polyethism drives community structure of the bacterial gut microbiota in the fungus-cultivating termite *Odontotermes formosanus*. Environ Microbiol 2016;18:1440–51. 10.1111/1462-2920.13046 26346907

[31] Otani S , Hansen LH, Sørensen SJ et al. Bacterial communities in termite fungus combs are comprised of consistent gut deposits and contributions from the environment. Microb Ecol 2016;71:207–20. 10.1007/s00248-015-0692-6 26518432 PMC4686563

[32] Eggleton P . An introduction to termites: biology, taxonomy and functional morphology. In: Bignell DE, Roisin Y, Lo N. et al. (eds.), Biology of Termites: A Modern Synthesis. Springer, Dordrecht, 1–26.

[33] Nobre T , Rouland-Lefèvre C, Aanen DK. Comparative Biology of Fungus Cultivation in Termites and Ants. In: Bignell DE, Roisin Y, Lo N. et al. (eds.), Biology of Termites: A Modern Synthesis. Springer, Dordrecht, 193–210.

[34] Poulsen M , Hu H, Li C et al. Complementary symbiont contributions to plant decomposition in a fungus-farming termite. Proc Natl Acad Sci USA 2014;111:14500–5. 10.1073/pnas.1319718111 25246537 PMC4209977

[35] Aanen DK , Eggleton P, Rouland-Lefèvre C et al. The evolution of fungus-growing termites and their mutualistic fungal symbionts. Proc Natl Acad Sci USA 2002;99:14887–92. 10.1073/pnas.222313099 12386341 PMC137514

[36] Aanen DK , Eggleton P. Fungus-growing termites originated in African rain forest. Curr Biol 2005;15:851–5. 10.1016/j.cub.2005.03.043 15886104

[37] Rouland-LeFèvre C , Diouf MN, Brauman A et al. Phylogenetic relationships in *Termitomyces* (family Agaricaceae) based on the nucleotide sequence of its: a first approach to elucidate the evolutionary history of the symbiosis between fungus-growing termites and their fungi. Mol Phyl Evol 2002;22:423–9 10.1006/mpev.2001.1071 11884167

[38] Roberts EM , Todd CN, Aanen DK et al. Oligocene termite nests with in situ fungus gardens from the Rukwa rift basin, Tanzania, support a paleogene African origin for insect agriculture. PLoS One 2016;11:e0156847. 10.1371/journal.pone.0156847 27333288 PMC4917219

[39] Nobre T , Fernandes C, Boomsma JJ et al. Farming termites determine the genetic population structure of *Termitomyces* fungal symbionts. Mol Ecol 2011;20:2023–33. 10.1111/j.1365-294X.2011.05064.x 21410808

[40] South EJ, Krishna K, Grimaldi DA et al. SF Isoptera: Isoptera Species File (version January 2018). In: Roskov Y, Ower G, Orrell T et al. eds. Species 2000 & ITIS Catalogue of Life, 2019 Annual Checklist. 2019. Digital resource at www.catalogueoflife.org/annual-checklist/2019. Species 2000: Naturalis, Leiden, the Netherlands.

[41] da Costa RR , Hu H, Li H et al. Symbiotic plant biomass decomposition in fungus-growing termites. Insects 2019;10:87. 10.3390/insects10040087 PMC6523192 30925664

[42] da Costa RR , Hu H, Pilgaard B et al. Enzyme activities at different stages of plant biomass decomposition in three species of fungus growing termites. Appl Environ Micr 2018;84:e01815-17. 10.1128/AEM.01815-17 PMC5812949 29269491

[43] Hu H , da Costa RR, Pilgaard B et al. Fungiculture in termites is associated with a mycolytic gut bacterial community. mSphere 2019;4:00165-19. 10.1128/mSphere.00165-19 PMC6520439 31092601

[44] Otani S , Challinor VL, Kreuzenbeck NB et al. Disease-free monoculture farming by fungus-growing termites. Sci Rep 2019;9:8819. 10.1038/s41598-019-45364-z 31217550 PMC6584615

[45] Bodawatta KH , Poulsen M, Bos N. Foraging *Macrotermes natalensis* fungus-growing termites avoid a mycopathogen but not an entomopathogen. Insects 2019;10:185. 10.3390/insects10070185 31247889 PMC6681374

[46] Katariya L , Ramesh PB, Gopalappa T et al. Fungus-farming termites selectively bury weedy fungi that smell different from crop fungi. J Chem Ecol 2017;43:986–95. 10.1007/s10886-017-0902-4 29124530

[47] Katariya L , Ramesh PB, Sharma A et al. Local hypoxia generated by live burial is effective in weed control within termite fungus farms. Insect Soc 2018;65:561–9. 10.1007/s00040-018-0644-5

[48] Katariya L , Ramesh PB, Borges RM. Dynamic environments of fungus-farming termite mounds exert growth-modulating effects on fungal crop parasites. Environ Microbiol 2018;20:971–9. 10.1111/1462-2920.14026 29235709

[49] Schmidt S , Bos N, Murphy R et al. Make the environment protect you from disease: elevated CO_2_ inhibits antagonists of the fungus-farming termite symbiosis. Front Ecol Evo*l* 2023;11:1134492. 10.3389/fevo.2023.1134492

[50] Beemelmanns C , Ramadhar TR, Kim KH et al. Macrotermycins a-d, glycosylated macrolactams from a termite-associated *Amycolatopsis* sp. M39. Org Lett 2017;19:1000–3. 10.1021/acs.orglett.6b03831 28207275 PMC6006516

[51] Beemelmanns C , Guo H, Rischer M et al. Natural products from microbes associated with insects. Beilstein J Org Chem 2016;12:314–27. 10.3762/bjoc.12.34 26977191 PMC4778507

[52] Murphy R , Benndorf R, de Beer ZW et al. Comparative genomics reveals prophylactic and catabolic capabilities of Actinobacteria within the fungus-farming termite symbiosis. mSphere 2021;6:e01233–20. 10.1128/msphere.01233-20 33658277 PMC8546716

[53] Schmidt S , Kildgaard S, Guo H et al. The chemical ecology of the fungus-farming termite symbiosis. Nat Prod Rep 2022;39:231–48. 10.1039/D1NP00022E 34879123 PMC8865390

[54] Yin C , Jin L, Li S et al. Diversity and antagonistic potential of Actinobacteria from the fungus-growing termite *Odontotermes formosanus*. 3 Biotech 2019;9:45. 10.1007/s13205-019-1573-3 PMC6342738 30729069

[55] Lee SR , Lee D, Yu JS et al. Natalenamides a–c, cyclic tripeptides from the termite-associated *Actinomadura* sp. Rb99 Molecules 2018;23:3003. 10.3390/molecules23113003 30453579 PMC6278286

[56] Benndorf R , Guo H, Sommerwerk E et al. Natural products from Actinobacteria associated with fungus-growing termites. Antibiotics 2018;7:83. 10.3390/antibiotics7030083 30217010 PMC6165096

[57] Um S , Fraimout A, Sapountzis P et al. The fungus-growing termite *Macrotermes natalensis* harbors bacillaene-producing *Bacillus* sp. that inhibit potentially antagonistic fungi. Sci Rep 2013;3:1–7 10.1038/srep03250 PMC3832938 24248063

[58] Wyche TP , Ruzzini AC, Beemelmanns C et al. Linear peptides are the major products of a biosynthetic pathway that encodes for cyclic depsipeptides. Org Lett 2017;19:1772–5. 10.1021/acs.orglett.7b00545 28326787 PMC6013059

[59] Carr G , Poulsen M, Klassen JL et al. Microtermolides a and b from termite-associated *Streptomyces* sp. and structural revision of vinylamycin. Org Lett 2012;14:2822–5. 10.1021/ol301043p 22591554 PMC3365539

[60] Diouf M , Hervé V, Fréchault S et al. Succession of the microbiota in the gut of reproductives of *Macrotermes subhyalinus* (Termitidae) at colony foundation gives insights into symbionts transmission. Front Ecol Evol 2023;10:1055382. 10.3389/fevo.2022.1055382

[61] Sinotte VM , Renelies-Hamilton J, Andreu-Sánchez S et al. Selective enrichment of founding reproductive microbiomes allows extensive vertical transmission in a fungus-farming termite. Proc R Soc B 2023;290:20231559. 10.1098/rspb.2023.1559 PMC10581767 37848067

[62] Huxley MM , Romano M, Edith MM et al. Fungal diversity and community structure in gut, mound and surrounding soil of fungus-cultivating termites. Afr J Microbiol Res 2017;11:504–15. 10.5897/AJMR2017.8484

[63] Vidkjær NH , Schmidt S, Hu H et al. Species- and caste-specific gut metabolomes in fungus-farming termites. Meta 2021;11:839. 10.3390/metabo11120839 PMC8707012 34940597

[64] Conlon BH , Schmidt S, Poulsen M et al. Orthogonal protocols for DNA extraction from filamentous fungi. STAR Protocols 2022;3:101126. 10.1016/j.xpro.2022.101126 35112085 PMC8790498

[65] Zaman M , Khan IA, Schmidt S et al. Morphometrics, distribution, and DNA barcoding: an integrative identification approach to the genus *Odontotermes* (termitidae: Blattodea) of Khyber Pakhtunkhwa, Pakistan. Forests 2022;13:674. 10.3390/f13050674

[66] Ayuso-Sacido A , Genilloud O. New PCR primers for the screening of NRPS and PKS-I systems in actinomycetes: detection and distribution of these biosynthetic gene sequences in major taxonomic groups. Microb Ecol 2005;49:10–24. 10.1007/s00248-004-0249-6 15614464

[67] Klindworth A , Pruesse E, Schweer T et al. Evaluation of general 16s ribosomal RNA gene PCR primers for classical and next-generation sequencing-based diversity studies. Nucl Acids Res 2013;41:e1. 10.1093/nar/gks808 22933715 PMC3592464

[68] Bolyen E , Rideout JR, Dillon MR et al. Reproducible, interactive, scalable and extensible microbiome data science using qiime 2. Nature Biotech 2019;37:852–7. 10.1038/s41587-019-0209-9 PMC7015180 31341288

[69] Martin M. Cutadapt removes adapter sequences from high-throughput sequencing reads. EMBnet.journal, 2011;17:10–12. 10.14806/ej.17.1.200

[70] Callahan BJ , McMurdie PJ, Rosen MJ et al. Dada2: high-resolution sample inference from illumina amplicon data. Nature Meth 2016;13:581–3. 10.1038/nmeth.3869 PMC4927377 27214047

[71] Bokulich NA , Kaehler BD, Rideout JR et al. Optimizing taxonomic classification of marker-gene amplicon sequences with qiime 2’s q2-feature-classifier plugin. Microbiome 2018;6:90. 10.1186/s40168-018-0470-z 29773078 PMC5956843

[72] Quast C , Pruesse E, Yilmaz P et al. The Silva ribosomal RNA gene database project: improved data processing and web-based tools. Nucl Acids Res 2013;41:D590–6. 10.1093/nar/gks1219 23193283 PMC3531112

[73] Robeson MS , O’Rourke DR, Kaehler BD et al. Rescript: reproducible sequence taxonomy reference database management. PLoS Comput Biol 2021;17:e1009581. 10.1371/journal.pcbi.1009581 34748542 PMC8601625

[74] McMurdie PJ , Holmes S. Phyloseq: an r package for reproducible interactive analysis and graphics of microbiome census data. PLoS One 2013;8:e61217. 10.1371/journal.pone.0061217 23630581 PMC3632530

[75] Team RC R . A Language and Environment for Statistical Computin*g*. Austria: Vienna.

[76] Davis NM , Proctor DM, Holmes SP et al. Simple statistical identification and removal of contaminant sequences in marker-gene and metagenomics data. Microbiome 2018;6:226. 10.1186/s40168-018-0605-2 30558668 PMC6298009

[77] Oksanen FJ, et al. Vegan: Community Ecology Package. R package Version 2.4-3. https://CRAN.R-project.org/package=vegan. 2017.

[78] Ben-Shachar M , Lüdecke D, Makowski D. Effect size: estimation of effect size indices and standardized parameters. JOSS 2020;5:2815. 10.21105/joss.02815

[79] Wickham H. 2016. ggplot2: Elegant Graphics for Data Analysis. Springer-Verlag New York. ISBN 978-3-319-24277-4. https://ggplot2.tidyverse.org

[80] Kurtz ZD , Müller CL, Miraldi ER et al. Sparse and compositionally robust inference of microbial ecological networks. PLoS Comput Biol 2015;11:e1004226. 10.1371/journal.pcbi.1004226 25950956 PMC4423992

[81] Müller CL , Bonneau R, Kurtz Z. Generalized Stability Approach for Regularized Graphical Model*s*, 2016. https://arxiv.org/abs/1605.07072

[82] Liu H , Roeder K, Wasserman L. Stability approach to regularization selection (stars) for high dimensional graphical models. Adv Neural Inf Process Syst 2010;24:1432–40. 25152607 PMC4138724

[83] Sievers F , Wilm A, Dineen D et al. Fast, scalable generation of high-quality protein multiple sequence alignments using clustal omega. Mol Syst Biol 2014;7:539–9. 10.1038/msb.2011.75 PMC3261699 21988835

[84] Terlouw BR , Blin K, Navarro-Muñoz JC et al. Mibig 3.0: a community-driven effort to annotate experimentally validated biosynthetic gene clusters. Nucl Acids Res 2023;51:D603–10. 10.1093/nar/gkac1049 36399496 PMC9825592

[85] Camacho C , Coulouris G, Avagyan V et al. Blast+: architecture and applications. BMC Bioinf 2009;10:421. 10.1186/1471-2105-10-421 PMC2803857 20003500

[86] Yang X , Yousef AE. Antimicrobial peptides produced by *Brevibacillus* spp.: structure, classification and bioactivity: a mini review. World J Microbiol Biotechnol 2018;34:57. 10.1007/s11274-018-2437-4 29594558

[87] Klassen JL , Lee SR, Poulsen M et al. Efomycins k and l from a termite-associated *Streptomyces* sp. M56 and their putative biosynthetic origin. Front Microbiol 2019;10:01739. 10.3389/fmicb.2019.01739 PMC6691879 31447803

[88] Guo H , Benndorf R, Leichnitz D et al. Isolation, biosynthesis and chemical modifications of rubterolones a–f: rare tropolone alkaloids from *Actinomadura* sp. 5-2. Chemistry - A Eur J 2017;23:9338–45. 10.1002/chem.201701005 28463423

[89] Kim KH , Ramadhar TR, Beemelmanns C et al. Natalamycin a, an ansamycin from a termite-associated *Streptomyces* sp. Chem Sci 2014;5:4333–8. 10.1039/C4SC01136H 25386334 PMC4224317

[90] Faust K , Sathirapongsasuti JF, Izard J et al. Microbial co-occurrence relationships in the human microbiome. PLoS Comput Biol 2012;8:e1002606. 10.1371/journal.pcbi.1002606 22807668 PMC3395616

[91] Hernandez DJ , David AS, Menges ES et al. Environmental stress destabilizes microbial networks. ISME J 2021;15:1722–34. 10.1038/s41396-020-00882-x 33452480 PMC8163744

[92] Tracanna V , Ossowicki A, Petrus MLC et al. Dissecting disease-suppressive rhizosphere microbiomes by functional amplicon sequencing and 10× metagenomics. mSystems 2021;6:01116–20. 10.1128/msystems.01116-20 PMC8269251 34100635

[93] Wang X , Zhou H, Chen H et al. Discovery of recombinases enables genome mining of cryptic biosynthetic gene clusters in Burkholderiales species. Proc Natl Acad Sci USA 2018;115:E4255–63. 10.1073/pnas.1720941115 29666226 PMC5939090

[94] Rautenbach M , Troskie AM, Vosloo JA et al. Antifungal membranolytic activity of the tyrocidines against filamentous plant fungi. Biochimie 2016;130:122–31. 10.1016/j.biochi.2016.06.008 27328781

[95] Visser AA , Ros VID, De Beer ZW et al. Levels of specificity of Xylaria species associated with fungus-growing termites: a phylogenetic approach. Mol Ecol 2009;18:553–67. 10.1111/j.1365-294x.2008.04036.x 19161474

[96] Visser AA , Kooij PW, Debets AJM et al. *Pseudoxylaria* as stowaway of the fungus-growing termite nest: interaction asymmetry between *Pseudoxylaria, Termitomyces* and free-living relatives. Fun Ecol 2011;4:322–32 10.1016/j.funeco.2011.05.003

[97] Leuthold RH , Badertscher S, Imboden H. The inoculation of newly formed fungus comb with termitomyces in *Macrotermes* colonies (Isoptera, Macrotermitinae). Insect Soc 1989;36:328–38. 10.1007/BF02224884

[98] Badertscher S , Gerber C, Leuthold RH. Polyethism in food supply and processing in termite colonies of *Macrotermes subhyalinus* (isoptera). Behav Ecol Sociobiol 1983;12:115–9. 10.1007/BF00343201

