# Supplementary material for: Non-ribosomal peptide synthase profiles remain structurally similar despite minimally shared features across fungus-farming termite microbiomes
Source: ISME Commun. 2024 Jul 11;4(1):ycae094. doi: 10.1093/ismeco/ycae094 (PMC11789546; doi:10.1093/ismeco/ycae094)
Supplement: Supplemental_Information_ycae094 [file supplemental_information_ycae094.docx]

**Supplemental Material for**

**Non-Ribosomal Peptide Synthase profiles remain structurally similar despite minimally shared features across fungus-farming termite microbiomes**

Robert Murphy^a^, Mikael Lenz Strube^b^, Simon Kolotchéléma Silue^c,d^, N’golo A. Koné^c,d^, Søren Rosendahl^a^, and Michael Poulsen^a^

**Supplemental Figures with legends**

**Figure S1**


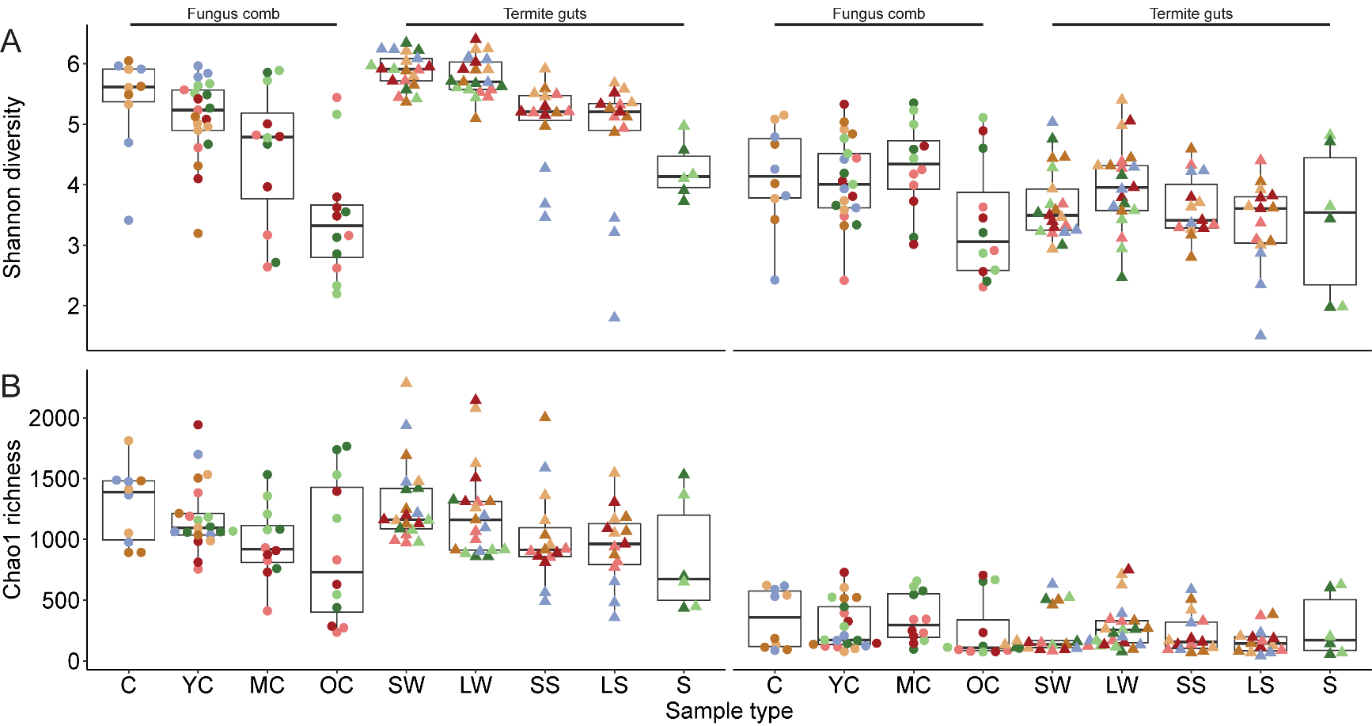


**Figure S1.** ASV and OBUs alpha diversities across the fungus-farming termite symbiosis. **A**: Chao 1 richness of ASVs and OBUs, C = mixed comb, YC = young comb, MC = mature comb, OC = old comb, SW = small worker, LW = large worker, SS = small soldier, LS = large soldier, S = soldier (for *Odontotermes* that only has one soldier caste). **B:** Shannon richness of ASV and OBUs with the same labelling scheme as panel A.

**Figure S2**


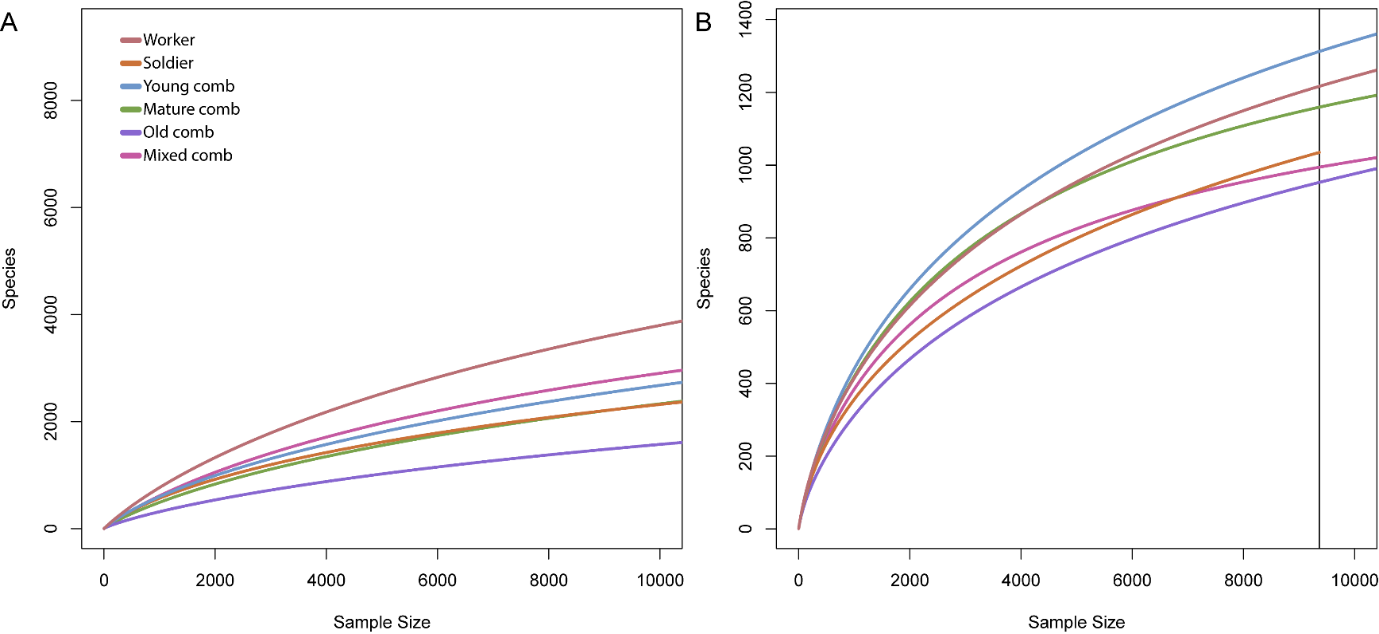


**Figure S2.** Saturation of biosynthetic potential and taxonomic diversity was achieved in all sample types. **A:** Rarefaction curves for ASVs showing observed features by sample types (averaged across termite species). **B:** Rarefaction curves for OBUs showing observed features by sample type (averaged across termite species).

**Supplemental Table Legends**

**Table S1.** Overview of all samples collected and their associated metadata. Available as separate document.

**Table S2.** TukeyHSD results of ANOVA tests of the effect of sample type, termite host species and their interaction on ASV (top) and OBU (bottom) Shannon diversity. Available as separate document.

**Table S3.** Pairwise PERMANOVA results of 16S rRNA ASV composition tested against independent variables and their interactions. Distances are Bray-Curtis dissimilarity. Available as separate document.

**Table S4.** Pairwise PERMANOVA results of OBU compositions tested against independent variables and their interactions. Distances are Bray-Curtis dissimilarity. Available as separate document.

**Table S5.** Pairwise PERMANOVA results of 16S rRNA ASV compositions, grouped to genus, tested against independent variables and their interactions. Distances are Bray-Curtis dissimilarity. Available as separate document.

**Table S6.** Species core OBU relative abundances for termite guts and fungus combs. The “Sum/total” column shows the sum of relative abundance divided by percentage total OBUs in a species that the core OBUs make up. Available as separate document.

**Table S7:** BLASTX output for species core OBUs, the single total comb core OBU and previously identified specialised metabolites. Only BGCs with hits in the MIBiG database and a bit-score >50 and sequence.
